# Inversion of the X-ray restrained wavefunction equations: a first step towards the development of exchange–correlation functionals based on X-ray data

**DOI:** 10.1107/S1600576725004765

**Published:** 2025-07-25

**Authors:** Alessandro Genoni, Maurizio Sironi

**Affiliations:** a Dipartimento di Chimica, Materiali e Ingegneria Chimica ‘Giulio Natta’ Politecnico di Milano Via Mancinelli 7 20131Milano Italy; b Dipartimento di Chimica Università degli Studi di Milano Via Golgi 19 20133Milano Italy; Universidad de Oviedo, Spain

**Keywords:** quantum crystallography, X-ray restrained wavefunctions, X-ray constrained wavefunctions, density functional theory, exchange–correlation functionals, electron density, orbital-averaged potentials

## Abstract

The X-ray restrained wavefunction (XRW) method is a quantum crystallographic technique that enables the determination of wavefunctions compatible with experimental X-ray diffraction data. Extensive research has demonstrated that this strategy inherently captures electron correlation and polarization effects on the electron density, while also providing consistent electron distributions. These findings suggest that the approach could be valuable in the development of new exchange–correlation (xc) functionals for density functional theory (DFT) calculations. This is particularly relevant in light of recent observations and recommendations by Medvedev *et al*. [*Science* (2017), **355**, 49–52], who stressed the importance that xc functionals give both accurate energy values and exact electron densities, in line with the original spirit of DFT. Motivated by this perspective, this paper presents a preliminary investigation that aims at extracting and visualizing for the first time the perturbation potentials arising from the use of X-ray diffraction data as restraints in XRW calculations. In the present work, these potentials are simply obtained as orbital-averaged potentials through straightforward inversions of the XRW equations, where theoretical or high-quality experimental X-ray structure factors are employed in XRW computations at the restricted Hartree–Fock level for atoms (neon, argon and krypton) and simple molecules (dilithium and urea). Features and limitations of the resulting preliminary potentials are illustrated, while future perspectives on the use of the XRW method for the development of xc functionals are also discussed.

## Abstract 2

The X-ray restrained wavefunction (XRW) approach could be a useful tool to propose new exchange–correlation (xc) functionals for density functional theory. Here, orbital-averaged XRW perturbation potentials of atoms (neon, argon and krypton) and simple molecules (dilithium and urea) are extracted and visualized for the first time, and their possible use in the development of new xc functionals is discussed.

## 1. Introduction

Quantum crystallography (Genoni, Bučinský *et al.*, 2018 ▸; Macchi, 2020 ▸, 2022 ▸; Krawczuk & Genoni, 2024 ▸) is an emerging field of science characterized by a strong and mutual connection between quantum physics and crystallography. In fact, if quantum physics (and particularly the laws of quantum mechanics) can be used to analyse and interpret the results of crystallographic experiments, accurate crystallographic measurements can also be exploited to reveal subatomic details of nature (Macchi, 2020 ▸, 2022 ▸). This connection is much stricter and older than scientists usually believe and dates back to the early days of quantum physics, when physicists and chemists immediately understood and envisaged the potential of the then recently discovered X-ray diffraction technique to unveil the distribution of electrons in atoms, molecules and solids, and to investigate the features of chemical bonds (Debye, 1915 ▸; Compton, 1915 ▸). At the same time, quantum mechanics was exploited to interpret the measured scattered intensities.

Today, quantum crystallography can also be considered as a research domain where the above-mentioned link between quantum physics and crystallography is highlighted and profitably exploited (Genoni, Bučinský *et al.*, 2018 ▸; Macchi, 2020 ▸, 2022 ▸; Krawczuk & Genoni, 2024 ▸). Examples in this direction are (i) the methods for the determination of electron-charge and spin-density distributions from experimental diffraction data [*e.g.* multipole model techniques (Stewart, 1976 ▸; Hansen & Coppens, 1978 ▸; Deutsch *et al.*, 2012 ▸, 2014 ▸) and maximum entropy strategies (Sakata & Sato, 1990 ▸; Roversi *et al.*, 1998 ▸; Van Smaalen & Netzel, 2009 ▸)]; (ii) the quantum chemical topological approaches for the analysis and interpretation of experimental or theoretical electron densities [*e.g.* quantum theory of atoms in molecules (Bader, 1990 ▸), interacting quantum atom strategy (Blanco *et al.*, 2005 ▸; Martín Pendás *et al.*, 2006 ▸; Pendás *et al.*, 2007 ▸, 2009 ▸; Tiana *et al.*, 2010 ▸; Martín Pendás *et al.*, 2023 ▸), noncovalent interaction index (Johnson *et al.*, 2010 ▸; Contreras-García *et al.*, 2011 ▸)]; (iii) *ab initio* methods to perform calculations on periodic systems, such as those implemented in well known suites of programs for solid state materials, such as *CRYSTAL* (Erba *et al.*, 2023 ▸; Dovesi *et al.*, 2022 ▸), *Quantum Espresso* (Giannozzi *et al.*, 2017 ▸; Carnimeo *et al.*, 2023 ▸) and *Wien2K* (Blaha *et al.*, 2020 ▸); and (iv) techniques characterized by a strong interplay between traditional methods and concepts of quantum chemistry and the results of diffraction/scattering experiments (Grabowsky *et al.*, 2017 ▸, 2020 ▸; Genoni & Macchi, 2020 ▸). The last group of strategies further subdivides into two subclasses: the subgroup of approaches in which diffraction data (not necessarily only X-ray diffraction but also polarized neutron diffraction, Compton scattering and magnetic Compton scattering data) are directly used in quantum chemistry calculations or along with quantum chemical concepts to obtain compatible ‘experimental’ one-electron reduced density matrices (Clinton & Massa, 1972 ▸; Massa *et al.*, 1985 ▸; Gueddida, Yan & Gillet, 2018 ▸; Gueddida, Yan, Kibalin *et al.*, 2018 ▸; De Bruyne & Gillet, 2020 ▸; Launay & Gillet, 2021 ▸; Matta & Massa, 2022 ▸; Yu & Gillet, 2024 ▸) or wavefunctions (Genoni, 2024 ▸); and the subgroup of strategies where, conversely, the quantum chemical calculations are exploited in crystal structure determinations to try to get better structural models, as is carried out in Hirshfeld atom refinements (Jayatilaka & Dittrich, 2008 ▸; Capelli *et al.*, 2014 ▸; Woińska *et al.*, 2014 ▸; Fugel, Jayatilaka *et al.*, 2018 ▸; Chodkiewicz *et al.*, 2020 ▸, Kleemiss *et al.*, 2021 ▸).

This paper will consider the X-ray restrained wavefunction (XRW) method (Genoni, 2024 ▸), a fully quantum crystallographic technique first introduced by Dylan Jayatilaka in 1998 (Jayatilaka, 1998 ▸). Today, it is widely recognized as the most advanced approach for obtaining wavefunctions consistent with experimental X-ray diffraction data. At first glance, obtaining a wavefunction from experimental X-ray structure factors might seem unconventional and impractical, since it involves using the results of experimental measurements to derive a purely theoretical construct. However, upon closer examination, it becomes evident that this technique is deeply rooted in quantum physics and quantum chemistry. In fact, the wavefunction, which encapsulates all possible information about a system according to quantum mechanics, can be considered as the most accurate model for describing physical systems at the microscopic level, making it ideal for also incorporating experimental data. Moreover, the Hohenberg & Kohn theorem (Hohenberg & Kohn, 1964 ▸) of density functional theory (DFT) establishes a one-to-one correspondence between ground-state wavefunctions and ground-state electron densities, and the close relationship between electron densities and experimental X-ray diffraction data further ensures the feasibility of the XRW approach. This also highlights another key motivation behind the development of the Jayatilaka technique: the possibility to gain deeper insights into the Hohenberg & Kohn mapping between ground-state electron densities and wavefunctions in many-electron systems, an aspect closely tied to the preliminary investigation reported in this work.

Although initially developed only in the restricted Hartree–Fock case (Jayatilaka, 1998 ▸; Jayatilaka & Grimwood, 2001 ▸; Grimwood & Jayatilaka, 2001 ▸; Bytheway, Grimwood & Jayatilaka, 2002 ▸; Bytheway, Grimwood, Figgis *et al.*, 2002 ▸; Grimwood *et al.*, 2003 ▸), the XRW method has been gradually extended over the years. For instance, today the approach can also be used to treat open-shell systems within the unrestricted Hartree–Fock formalism (Hudák *et al.*, 2010 ▸), to extract extremely localized molecular orbitals from experimental X-ray diffraction data (Genoni, 2013*a* ▸,*b* ▸; Dos Santos *et al.*, 2014 ▸; Genoni & Meyer, 2016 ▸), to investigate relativistic effects on electron distributions of transition metal complexes through combination with quantum chemical relativistic methods (Hudák *et al.*, 2010 ▸; Bučinský *et al.*, 2016 ▸), and to extract traditional and useful chemical information (*e.g.* resonance structure weights) from X-ray diffraction experiments by relying on multi-determinant wavefunction *Ansätze* strongly rooted in valence bond theory (Genoni, 2017 ▸; Casati *et al.*, 2017 ▸; Genoni, Franchini *et al.*, 2018 ▸; Genoni, Macetti *et al.*, 2019 ▸).

Beyond the extensive methodological advancements mentioned in the previous paragraph, since its inception the Jayatilaka method has also been widely and successfully applied to investigate various chemical and physical phenomena/problems. It has proven especially valuable in the study of chemical bonding, for example by aiding in the interpretation of chemical reactivities (Grabowsky *et al.*, 2010 ▸, 2011 ▸), by rationalizing hypervalency and resonance issues (Grabowsky *et al.*, 2012 ▸; Fugel, Malaspina *et al.*, 2019 ▸; Fugel, Kleemiss *et al.*, 2018 ▸; Fugel, Ponomarenko *et al.*, 2019 ▸), and by analysing and quantifying crucial noncovalent interactions in compounds of pharmaceutical interest (Thomas, Satheeshkumar *et al.*, 2015 ▸; Thomas, Jayatilaka & Guru-Row, 2015 ▸; Singh *et al.*, 2024 ▸). The XRW strategy has also been exploited in determining optoelectronic properties (Whitten *et al.*, 2006 ▸; Jayatilaka *et al.*, 2009 ▸; Hickstein *et al.*, 2013 ▸; Cole & Hickstein, 2013 ▸). In this context, the technique was specifically applied in studies focused on the determination of dipole moments, polarizabilities, hyperpolarizabilities and refractive indices in molecules exhibiting significant non-linear optical activities. These investigations also revealed, for the first time, the inherent ability of the Jayatilaka method to incorporate polarization effects caused by the presence of neighbouring molecular units within the crystal.

The last aspect has also been the primary focus of a series of dedicated investigations that have empirically and conclusively demonstrated the intrinsic capability of the XRW approach to capture both electron correlation and polarization effects on the electron density. In an initial study, this was proven only for electron correlation by using theoretical X-ray structure factors derived from gas-phase CCSD (coupled cluster with single and double excitations) calculations (Genoni, Dos Santos *et al.*, 2017 ▸). Later, Ernst *et al.* (2020 ▸) applied the same strategy to examine polarization effects but also made the first use of experimental X-ray diffraction data in these types of studies. Finally, Hupf *et al.* (2023 ▸) independently used both theoretical and experimental X-ray structure factors to conduct a comprehensive analysis of electron correlation and polarization, and examined the effects not only on electron-density distributions but also on an exchange–correlation (xc) potential commonly used in DFT calculations [namely, the BLYP (Becke–Lee–Yang–Parr) xc potential]. Despite these results, the influence of experimental uncertainties in the X-ray data employed in the XRW calculations remains an open question and will warrant more thorough analyses in future work.

The findings from previous studies have been further strengthened by a recent investigation that compared traditional gas-phase DFT computations with XRW calculations, both using the same xc functionals but incorporating experimental X-ray diffraction data as restraints in the latter. By primarily relying on topological properties of the electron density for the comparison, the study revealed that, regardless of the specific DFT xc functional used, the XRW computations produced more consistent electron-density results, mitigating the significant variability often seen in standard gas-phase calculations (Genoni & Martín Pendás, 2024 ▸). However, the same study also pointed out the need of introducing a completely periodic XRW technique to fully capture the influence of the crystalline environment and to completely disentangle the shortcomings of the different density functional approximations from those of the Jayatilaka strategy.

The inherent ability of the XRW approach to incorporate electron correlation and polarization effects while providing consistent electron-density distributions becomes especially important in light of a recent observation by Medvedev *et al.* (2017 ▸). They highlighted that, despite excellent energetic results, modern xc functionals often produce electron densities that significantly deviate from the ‘exact’ ones, thus drifting away from the original spirit of DFT. Therefore, in their well known and provocative paper, Medvedev and colleagues argued that future developments of xc functionals should focus not only on optimizing energy fittings and enforcing theoretical constraints but also on minimizing deviations from ‘exact’ electron densities (Medvedev *et al.*, 2017 ▸). To some extent, the point raised by Medvedev *et al.* and the central role played by the electron density was also recently highlighted by Skogh *et al.* (2024 ▸), who pointed out how electron distributions resulting from high-level *ab initio* computations or X-ray diffraction experiments could be exploited as benchmarks for the validation of quantum computations.

Given these considerations, the Jayatilaka method emerges as a potentially optimal tool for developing new xc functionals or refining already existing xc functionals commonly used in DFT calculations. Within this context, in this work, we present an initial attempt to extract and visualize the perturbation potentials that are automatically introduced in quantum chemical computations when X-ray diffraction data are employed as restraints. These perturbation potentials have not been analysed so far and can be potentially viewed as corrections to standard xc potentials, thus envisaging their future use as models or components in the design of new xc functionals [for example, as is done for HCTH xc functionals (Hamprecht *et al.*, 1998 ▸; Boese *et al.*, 2000 ▸; Boese & Handy, 2002 ▸)]. This could also open the way towards the development of xc functionals based on experimental X-ray diffraction data, a route that has not been considered until now, despite the strict relationship between X-ray structure factors and the basic variable of DFT (*i.e.* the electron density). However, the goal of the present paper is not to propose novel functionals but rather to start visualizing, for the first time, the perturbation potentials of the XRW method, and to possibly pave an alternative/original (quantum crystallographic) way in xc functional development. We also believe that the research perspective presented through this preliminary work shows a possible further synergy between quantum crystallography and one of its closest research domains, namely quantum chemistry.

The paper is organized as follows. In Section 2.1, the basic theory of the XRW approach will be briefly reviewed, especially in its original restricted Hartree–Fock version; in Section 2.2, the strategy to invert the XRW equations in the closed-shell case and to consequently obtain orbital-averaged XRW perturbation potentials will be illustrated and commented on; in Section 3.1, details on the investigated systems (neon, argon, krypton, dilithium and urea) and on the performed calculations will be given; in Section 3.2, the orbital-averaged XRW xc potentials and the orbital-averaged XRW perturbation potentials obtained through the inversion procedure introduced in Section 2.2 will be shown and analysed; finally, in Section 4, conclusions will be drawn and possible future perspectives for the proposed research direction will be discussed.

## 2. Theory

### 2.1. Fundamentals of the XRW approach

As mentioned in the *Introduction*[Sec sec1], the XRW technique was initially developed by Dylan Jayatilaka in 1998 (Jayatilaka, 1998 ▸). It was further refined in the early 2000s by Jayatilaka and his collaborators (Jayatilaka & Grimwood, 2001 ▸; Grimwood & Jayatilaka, 2001 ▸; Bytheway, Grimwood & Jayatilaka, 2002 ▸; Bytheway, Grimwood, Figgis *et al.*, 2002 ▸; Grimwood *et al.*, 2003 ▸), who also integrated it into the quantum crystallographic software *Tonto* (Jayatilaka & Grimwood, 2003 ▸). Although different research groups have proposed significant methodological extensions of the strategy over the years, the Hartree–Fock version remains the most widely used variant. This is also the version that we have chosen for the current investigation. Therefore, in this section, the XRW method will be mainly presented within the restricted Hartree–Fock formalism.

Before briefly reviewing the Jayatilaka approach, we note that the technique was originally introduced as the ‘X-ray constrained wavefunction’ (XCW) strategy. However, several researchers in the field of quantum crystallography have highlighted that the X-ray structure factors employed in the calculations act as restraints rather than constraints (Grabowsky *et al.*, 2017 ▸; Ernst *et al.*, 2020 ▸; Macetti *et al.*, 2021 ▸; Genoni, 2022 ▸). Consequently, the method has been renamed as the ‘X-ray restrained wavefunction’ (XRW) technique. This is the terminology that we have decided to adopt throughout this work, although readers should be aware that both terms, ‘X-ray restrained wavefunction’ (XRW) and ‘X-ray constrained wavefunction’ (XCW), are used in equivalent ways in the literature.

In all current versions of the XRW technique, the underlying hypothesis is to work with fictitious molecular crystals formed by non-interacting molecular units but characterized by a global electron density that is equivalent to the overall electron distribution of the corresponding real interacting system. It is also assumed that the different molecular units of the crystal are described by independent wavefunctions, which have the same analytical expression according to the selected *Ansatz* (*e.g.* single Slater determinant wavefunction in the Hartree–Fock case) and which are related to each other through the symmetry operations of the crystal (namely, through the roto-translations 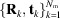, where 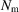 is the number of symmetry-equivalent positions in the crystal unit cell).

On the basis of the previous hypotheses, the electron density of the crystal unit cell can be written as follows:
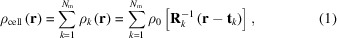
namely, only in terms of the electron density 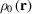 of the reference molecular unit, which is associated with the wavefunction 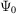 describing the same molecule. For the sake of completeness, we also emphasize that the XRW technique is currently applicable only to molecular crystals, and its extension to extended solids remains an open area of research.

In the current implementations of the XRW method, the wavefunction 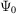 is obtained through the minimization of the ‘Jayatilaka functional’ (regardless of the analytical expression for 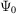):
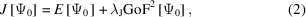
with *E* as the energy of the investigated system and 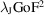 as a term that accounts for the perturbation introduced by the X-ray diffraction data directly used in the calculations. This additional term allows the global electron density for the fictitious non-interacting crystal to be as close as possible to the overall electron density of the corresponding real interacting system. Moreover, the term 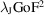 is given by two factors: (*a*) 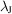, which is an external multiplier that is manually adjusted during the computations to tune the weight of the X-ray data (see below for further comments on the meaning and determination of 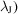;^1^ and (*b*) the square of the goodness of fit (GoF^2^), which can be considered as a measure of the statistical agreement between the calculated and target (generally experimental) structure-factor amplitudes:^2^
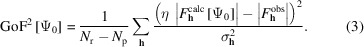
In equation (3), 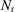 is the number of considered structure-factor amplitudes, 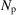 is the number of adjustable parameters, 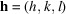 is the triad of Miller indices associated with the reflection and 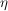 is an **h**-independent scale factor that puts the calculated structure-factor amplitudes 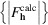 on the same scale as the target ones 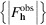, which also come along with a set of experimental uncertainties 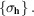


For the following derivation, we introduce the total scattering operator 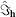 for a 2*N*-electron system:
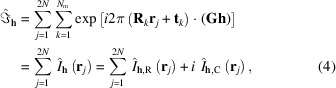
where **G** is the metric tensor of the reciprocal lattice and 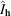 is the one-electron scattering operator (with 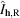 and 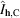 as its real and imaginary parts, respectively). This scattering operator is fundamental for the computation of the calculated structure-factor amplitudes.

So far, the analytical form of the reference crystal unit wavefunction has not been specified, so the previous theoretical aspects are common to all the existing variants of the XRW technique. However, as mentioned above, in this work we will only consider the restricted Hartree–Fock case and, therefore, we will hereafter assume 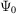 as having the form of the following single Slater determinant:
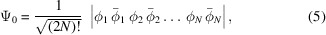
with each generic spatial orbital 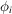 doubly occupied [namely, in equation (5), 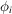 denotes a spatial orbital paired with a spin function 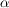, while 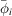 indicates a spatial orbital paired with a spin function 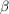].

By exploiting equation (4) and assuming a wavefunction *Ansatz* corresponding to equation (5), it can be easily proven that searching for the Slater determinant wavefunction 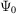 that minimizes the Jayatilaka functional 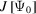 is equivalent to solving the following set of modified restricted Hartree–Fock equations:
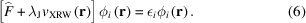

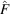
 is the traditional Fock operator of quantum chemistry given by
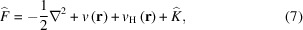
where 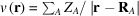 is the external potential due to the attraction between nuclei and electrons, 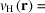

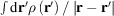
 is the Hartree potential due to the Coulomb electron repulsion [with 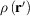 as the electron density of the system under examination], and 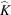 is the exchange operator defined as
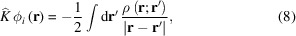
with the density matrix 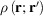 given by
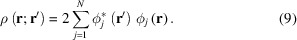
Furthermore, by considering equation (6), the XRW potential 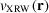 (tuned by the external multiplier 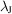) can be expressed as follows:
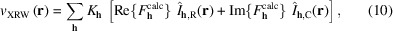
where the **h**-dependent constant 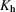 is
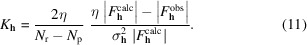
Therefore, the additional term 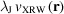 in equation (6) can be interpreted as the perturbation to the Fock operator due to the introduction of X-ray diffraction data as restraints in the calculations.

Note that the XRW approach has also been extended to DFT. In this case, the Jayatilaka functional is expressed only in terms of the electron density,
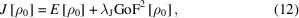
and the goal becomes equivalent to determining the electron distribution 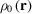 of the reference crystal unit that minimizes 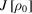. Working in the framework of the Kohn–Sham equations and assuming a 2*N*-electron closed-shell system, this is equivalent to solving the following set of modified Kohn–Sham equations:
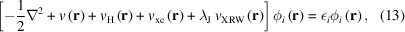
where 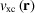 is the typical xc potential of the DFT calculations, while all the other terms have the same meanings and definitions as in equations (6) and (7). Furthermore, again in analogy with equation (6), the term 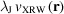 in equation (13) can be seen as a perturbation that is introduced when the X-ray diffraction data are directly used in the computations. However, in this case, it could also be interpreted as a correction to the approximations intrinsically contained in the xc potential associated with the adopted xc functional for the DFT calculation. This aspect further supports the possible exploitation of the Jayatilaka strategy in the development of new xc functionals.

It is also necessary to clarify the role of the 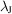 term. As previously mentioned, in all current implementations of the Jayatilaka technique, 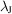 functions solely as an external multiplier that is manually adjusted to control the influence of X-ray data in the calculations, but it is not a Lagrange multiplier as originally described in the foundational papers on the XRW approach. As a result, determining the precise value of 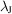 in XRW calculations remains an unresolved issue, despite the proposition of several criteria over the years [for a comprehensive discussion about this topic, readers are encouraged to consult a recent review on the XRW technique (Genoni, 2024 ▸)]. In general, the XRW computations are iterated until the maximum value of 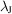 at which convergence is achieved, and this value is considered as the optimal one. However, it was recently proven that the correct value of 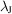 should be the maximum stationary point of the original version of the Jayatilaka functional (Genoni, 2022 ▸). This will give rise to a future reformulation and reimplementation of the technique, where the X-ray data will act as soft constraints (and no more as restraints) and where the 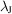 term will really have the meaning of a Lagrange multiplier.

### 2.2. Inversion of the XRW equations

As mentioned above, in this subsection we will consider the inversion of the X-ray restrained Hartree–Fock equations in the restricted case to determine orbital-averaged XRW perturbation potentials for closed-shell systems.

To accomplish this task, we rewrite the XRW equations in the Hartree–Fock case [see equations (6) and (7)] in the form of generalized Kohn–Sham equations:
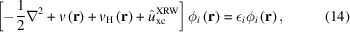
where
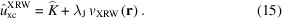
By multiplying equation (14) by 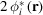 (to be done for each occupied molecular orbital), summing over the occupied molecular orbitals from 1 to *N* and dividing by the XRW electron density 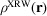, we obtain
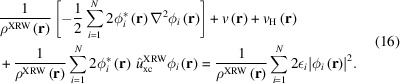
Now, the last term on the left-hand side of equation (16) can be defined as the ‘orbital-averaged XRW xc potential’:
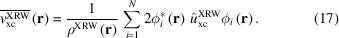
The right-hand side of equation (16) is hereafter defined as the ‘average local XRW orbital energy’:
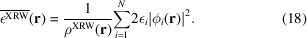
Also, the Laplacian form of the XRW kinetic energy density is given by
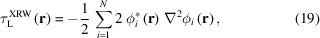
which can be rewritten as follows:
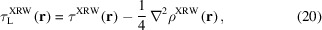
with
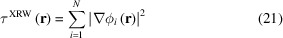
as the positive-definite form of the XRW kinetic energy density.

Substituting equations (17)–(21) into equation (16), we obtain the following expression:
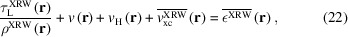
which can be reorganized as
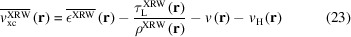
or, by exploiting equation (20), as
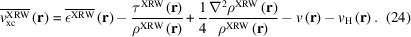
Equations (23) and (24) can be defined as the ‘XRW inversion formulae’ and are analogous to the ‘Kohn–Sham inversion formula’ introduced by Kananenka *et al.* (2013 ▸), which is the basis of a well known algorithm for the determination of xc potentials from given electron densities. As well as the above-mentioned approach by Kananenka and coworkers, other techniques have been proposed to obtain xc potentials compatible with electron distributions. Notable examples are the methods devised by Zhao *et al.* (1994 ▸), van Leeuwen & Baerends (1994 ▸) and Wu & Yang (2003 ▸). However, the investigation reported in this article is exclusively based on the strategy introduced by Kananenka and collaborators.

Equations (23) and (24) indicate that an orbital-averaged XRW xc potential can be simply obtained from an external potential 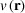, and from a set of molecular orbitals 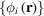 and a set of orbital energies 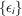 resulting from the resolution of the XRW equations [see equation (6)]. There are three possible cases:

(i) By using, as restraints, experimental X-ray structure-factor amplitudes resulting from a traditional X-ray diffraction experiment on a crystal, the orbital-averaged potential 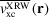 should account for xc effects plus the effects of the crystal environment (polarization effects) on the reference molecular unit.

(ii) By exploiting, as restraints, theoretically generated X-ray structure-factor amplitudes associated with a highly correlated wavefunction for an isolated molecule, the orbital-averaged potential 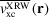 should include only exchange and correlation effects, thus indicating a new possible way of constructing xc potentials from high-level quantum chemical calculations.

(iii) By considering, as restraints, X-ray structure-factor amplitudes obtained from a single-molecule X-ray diffraction experiment based on recent developments and advancements in the field of X-ray free electron laser techniques (Odate *et al.*, 2023 ▸), we would obtain again an orbital-averaged potential 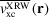 accounting for only exchange and correlation effects; however, in this case, we would open the possibility of constructing xc potentials from experimental and purely molecular electron densities.

Starting from equations (23) and (24), and following a procedure already proposed by Staroverov and coworkers (Kananenka *et al.*, 2013 ▸), it is also possible to determine orbital-averaged XRW perturbation potentials. They can be easily obtained as follows:
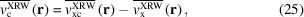
namely, as the difference of the two orbital-averaged potentials [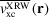 and 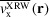] corresponding to the same electron density. The former results from the above-outlined XRW inversion procedure [see equations (23) or (24)] using self-consistent XRW molecular orbitals and orbital energies. The latter is obtained by substituting, in the inversion formula [see again equations (23) or (24)], the molecular orbitals and the orbital energies resulting from the diagonalization of the exchange-only Kohn–Sham Hamiltonian constructed with the self-consistent XRW molecular orbitals. This is the procedure that we adopted in this work to extract and start visualizing the (orbital-averaged) perturbation potentials of the XRW method, with the additional clarification that, in our case, the exchange-only Kohn–Sham Hamiltonian is the one constructed only with the Hartree–Fock exact exchange, namely the Hartree–Fock Hamiltonian.

As a caveat, we point out some common issues that typically affect orbital-averaged xc potentials like those discussed above. In fact, the described procedure is essentially a density-to-potential mapping, where an xc potential is fitted to a target density. Although this approach is formally rigorous, it encounters certain challenges when applied in practice:

(*a*) Small changes in the electron density can lead to significant variations in the xc potential, making the mapping problem ill-posed.

(*b*) In calculations involving finite basis sets (as in all our cases), the mapping from density to xc potential does not have a unique solution. As a result, the task of fitting an xc potential to a given density becomes ambiguous.

(*c*) Electron densities produced by Gaussian basis sets inherently result in xc potentials that exhibit oscillations near the atomic nuclei and diverge at long distances. In this work, we addressed this issue by introducing a smoothing technique developed by Staroverov and collaborators (Gaiduk *et al.*, 2013 ▸; Kananenka *et al.*, 2013 ▸). This method mitigates the basis set artefacts in the potential by subtracting the oscillatory components of the exchange-only local-density-approximation potential, previously calculated using the same basis set as that of the reference density.

Unfortunately, there are no easy solutions to overcome the first two drawbacks. However, the primary focus of this work is to visualize the perturbation potentials generated during XRW calculations. Our aim is to examine the features of these potentials and identify any emerging trends as the influence of the X-ray structure factors becomes more pronounced in the computations. At a later stage, we could imagine avoiding the numerical instabilities inherent in the proposed inversion strategy, for instance, by going beyond the simple density-to-potential mapping and combining the XRW method with much more robust techniques, such as the Ryabinkin–Kohout–Staroverov approach (Ryabinkin *et al.*, 2015 ▸; Cuevas-Saavedra *et al.*, 2015 ▸) and its modified version (Ospadov *et al.*, 2017 ▸).

## 3. Results and discussion

### 3.1. Computational details

To start visualizing the orbital-averaged XRW exchange–correlation and perturbation potentials obtained by inversion of the Jayatilaka equations, we opted to use structure-factor amplitudes obtained through both high-level quantum chemical calculations and accurate crystallographic experiments. The structure factors from the quantum chemical calculations were generated for small atomic and molecular systems, allowing us to analyse specific features of the extracted potentials based on data resolution and distance from atomic nuclei. On the other hand, the experimental structure factors demonstrated that this method is also fully applicable when high-quality charge-density X-ray diffraction data are available.

For the fully theoretical cases, we examined three atoms [neon (Ne), argon (Ar) and krypton (Kr)] and one small molecule, dilithium (Li_2_), with a bond distance set equal to 5.051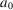 (the geometry is provided in the supporting information for completeness). For each of them, single-point calculations were initially performed at the CCSD level, using the universal Gaussian basis set (UGBS) for Ne and the uncontracted 6-311G* (u6-311G*) set of basis functions for the other three systems. The resulting electron densities were Fourier transformed analytically to obtain theoretical X-ray structure factors up to the desired resolutions. This was achieved by placing a single atom or molecule of each system in sufficiently large cubic unit cells to avoid interatomic or intermolecular interactions and using an in-house code that leverages Obara–Saika recurrence relations to compute Fourier transform integrals of Gaussian basis function products (Genoni, 2020 ▸). Details regarding the level of theory and basis set, the unit-cell size, the maximum resolution considered, and the total number of generated reflections for each case are summarized in Table 1 ▸. To assess the effects of the data resolution on the extracted potentials, we selected a higher maximum resolution value for the argon, krypton and dilithium systems. In these cases, to manage the increased number of associated reflections, we reduced the unit-cell size but always ensured the absence of interatomic or intermolecular interactions within the fictitious crystals.

The obtained theoretical X-ray structure-factor amplitudes were then used for XRW calculations. Since theoretical X-ray diffraction data were employed, we applied a recently introduced weighting scheme (Macetti & Genoni, 2023 ▸) with the weighting parameter 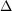 always set equal to 0.010 Å^−1^ to treat the structure factors more uniformly across different resolution ranges (see also the supporting information for more details); in fact, as noted in other studies (Genoni, Dos Santos *et al.*, 2017 ▸; Ernst *et al.*, 2020 ▸; Hupf *et al.*, 2023 ▸), theoretically generated X-ray data generally contain a much higher number of high-angle reflections compared with low- and medium-angle ones, which diminishes the significance of the latter. In the case of neon we did not notice any convergence issue, and we were thus able to perform the XRW calculations until the pre-established maximum value of 120.0 for the 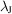 parameter. For argon and krypton, due to the much larger number of reflections used as restraints, the XRW computations were slower and their convergence more difficult. For this reason, we stopped the calculations at 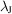 equal to 67.7 and 19.0 for Ar and Kr, respectively. Convergence problems were also observed for Li_2_, and, therefore, the XRW computations were iterated until 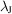 was equal to 26.7. The discussion on the trends of the extracted potential is independent of the 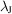 value at which the XRW calculations are halted. For completeness, we also indicate that, for all the XRW computations with theoretically generated X-ray diffraction data, the uncertainties 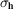 were set equal to 1.0.

In the experimental case, we carried out XRW calculations using the cc-pVTZ basis set and exploited the high-quality X-ray diffraction data obtained by Birkedal *et al.* (2004 ▸) for the urea crystal with a maximum resolution of 1.44 Å^−1^, a usual benchmark dataset for quantum crystallographic studies. We included all the deposited structure-factor amplitudes, excluding those with negative intensities, resulting in a total of 988 reflections (significantly fewer than in the computations with theoretical X-ray diffraction data discussed earlier). No weighting scheme was applied because a previous investigation has shown it to be ineffective when experimental structure factors are used in XRW calculations (Macetti & Genoni, 2023 ▸). The computations were iterated until the highest 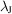 value for which the self-consistent field procedure converged (namely for 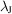 equal to 0.121) was reached. The XRW calculations were performed using the experimental in-crystal geometry of urea (which is planar; see the atomic coordinates provided in the supporting information) and the anisotropic displacement parameters (ADPs) resulting from the multipole model refinement of the collected X-ray data previously carried out by Birkedal *et al.* (2004 ▸). The ADPs were introduced in the XRW computations through the Stewart model (Stewart, 1969 ▸).

For all the XRW calculations (with theoretical or experimental X-ray structure factors), we used an internally modified version of *Gaussian 09* (Frisch *et al.*, 2009 ▸), where the working equations of the XRW approach were properly implemented.

Finally, the XRW molecular orbitals and orbital energies resulting from the above-mentioned calculations with theoretical and experimental structure-factor amplitudes were afterwards exploited to obtain orbital-averaged XRW exchange–correlation and perturbation potentials according to equations (23), (24) and (25), which have been previously implemented in a suitable in-house program.

### 3.2. Orbital-averaged XRW potentials

**Figure 1:**
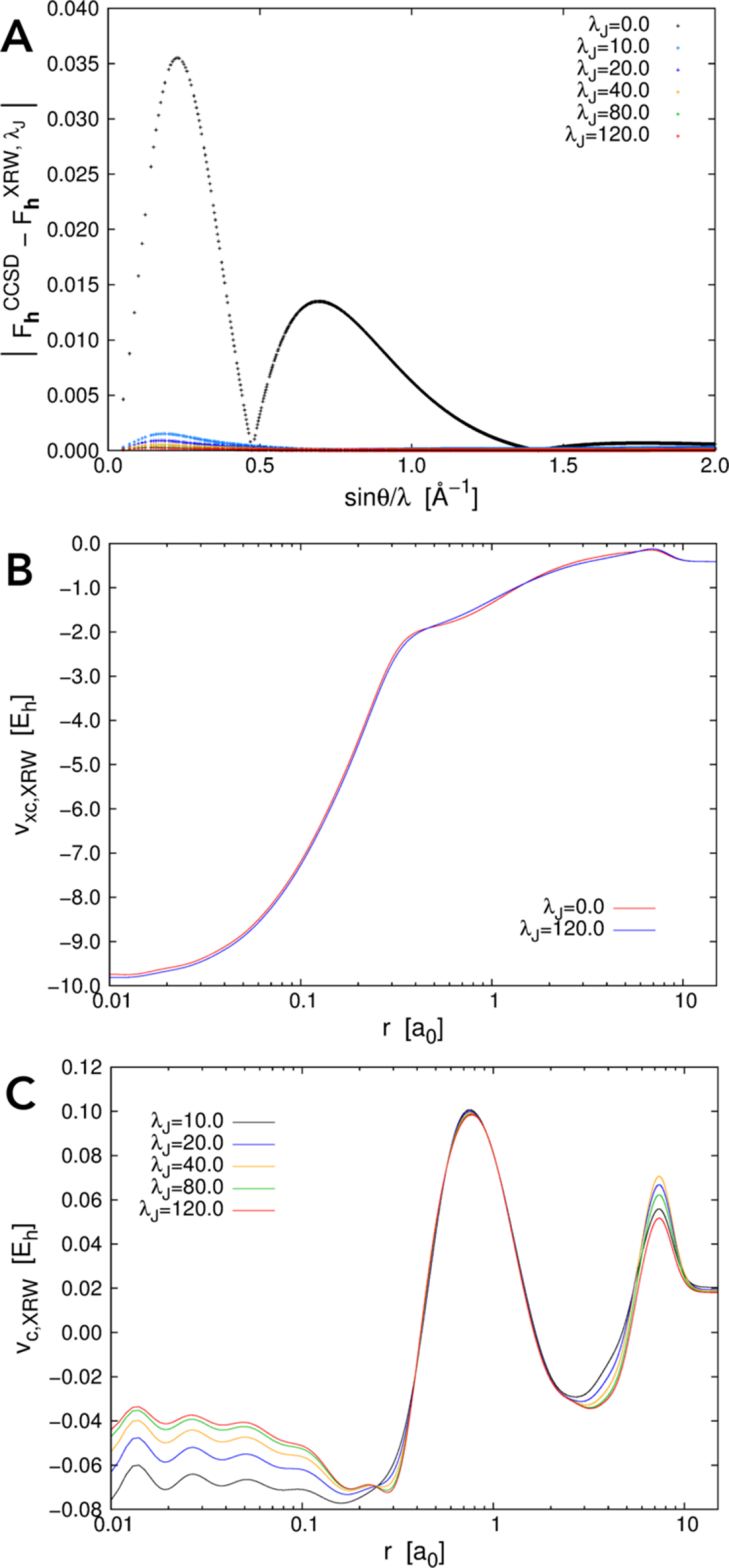
Neon: (*A*) absolute discrepancies between the XRW and CCSD/UGBS structure-factor amplitudes for different 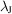 values, (*B*) orbital-averaged XRW xc potentials extracted for 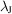 = 0.0 (Slater potential) and 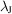 = 120.0, and (*C*) orbital-averaged XRW perturbation potentials obtained at different 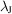 values.

Let us start by analysing the results based on the XRW calculations performed on the neon atom with theoretically generated X-ray structure factors. First, we highlight the fact that the discrepancies between the computed and the target CCSD/UGBS structure-factor amplitudes (and consequently the discrepancies between the corresponding electron densities) correctly and monotonically reduce as the external parameter 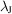 increases. This can be observed both in Table 2 ▸, where we report the global absolute error in the density, also used by Gould (2023 ▸) and defined as
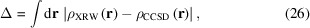
and in Fig. 1 ▸(*A*), where we depict the absolute deviations between the XRW and the CCSD structure-factor amplitudes for different 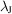 values. For completeness, in Fig. S1(*A*) of the supporting information we also show the variation of 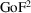 as a function of 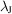.

Some of the orbital-averaged XRW potentials obtained for neon are shown in Fig. 1 ▸. In Fig. 1 ▸(*B*) we report the orbital-averaged XRW xc potentials extracted through the inversion procedure described in Section 2.2 for 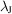 = 0.0 and 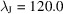, namely for the initial and final values of the external parameter. The former corresponds to the orbital-averaged potential resulting from a simple Hartree–Fock calculation, also known as the Slater potential. The two potentials show very similar trends and are only slightly different [refer to Fig. S2(*A*) for a graph illustrating the difference between the potentials], indicating that the introduction of the X-ray diffraction data in the calculations represents only a small perturbation to the original Hartree–Fock computation. This perturbation can be visualized more properly by considering the orbital-averaged XRW perturbation potentials depicted in Fig. 1 ▸(*C*) (see also the discussion in the next paragraph). These perturbation potentials closely resemble the difference between the orbital-averaged XRW xc potentials computed for 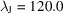 and 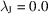 as shown in Fig. S2(*A*). However, the two sets of plots were generated using conceptually distinct approaches: (i) the perturbation potentials were derived via equation (25), by subtracting from the xc potentials at given 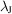 values the potentials obtained from simple diagonalizations of exchange-only Hamiltonians constructed with the converged XRW molecular orbitals at the same 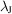 values; (ii) the difference plot in Fig. S2(*A*) was obtained by subtracting the Slater potential, constructed using molecular orbitals and orbital energies from a converged Hartree–Fock calculation (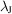 = 0), from the xc potential at 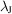 = 120.0.

By analysing the perturbation potentials in Fig. 1 ▸(*C*), we observe oscillations up to ∼0.25 Å from the nucleus. This can be attributed to the maximum resolution of 2.0 Å^−1^ for the X-ray structure-factor amplitudes used in the XRW calculations. In fact, on the basis of Bragg’s law, 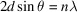, no data were available to model the potentials in regions very close to the nuclei. On the other hand, from ∼0.25 to ∼2.0 Å, the extracted perturbation potentials rapidly converge to a well defined shape, which can be explained by the fact that most of the reflections used as restraints in the computations fall in the resolution range 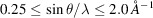. Between 2 and 6 Å, we observe again a gradual but much slower convergence in the extracted potentials, whereas beyond 6 Å, and particularly near the local maximum around 7 Å, the convergence becomes misaligned with respect to 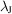. This can probably be explained by the significantly lower amount of X-ray data for 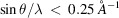 [see the number of points in this resolution range in Fig. 1 ▸(*A*)]. Additionally, unlike the cases of the other two atomic systems that will be analysed below (argon and krypton), the orbital-averaged XRW perturbation potentials of neon do not tend toward zero at large distances from the nucleus. At present, we do not have a definitive explanation for this behaviour, but we intend to explore it further in future studies.

**Figure 2:**
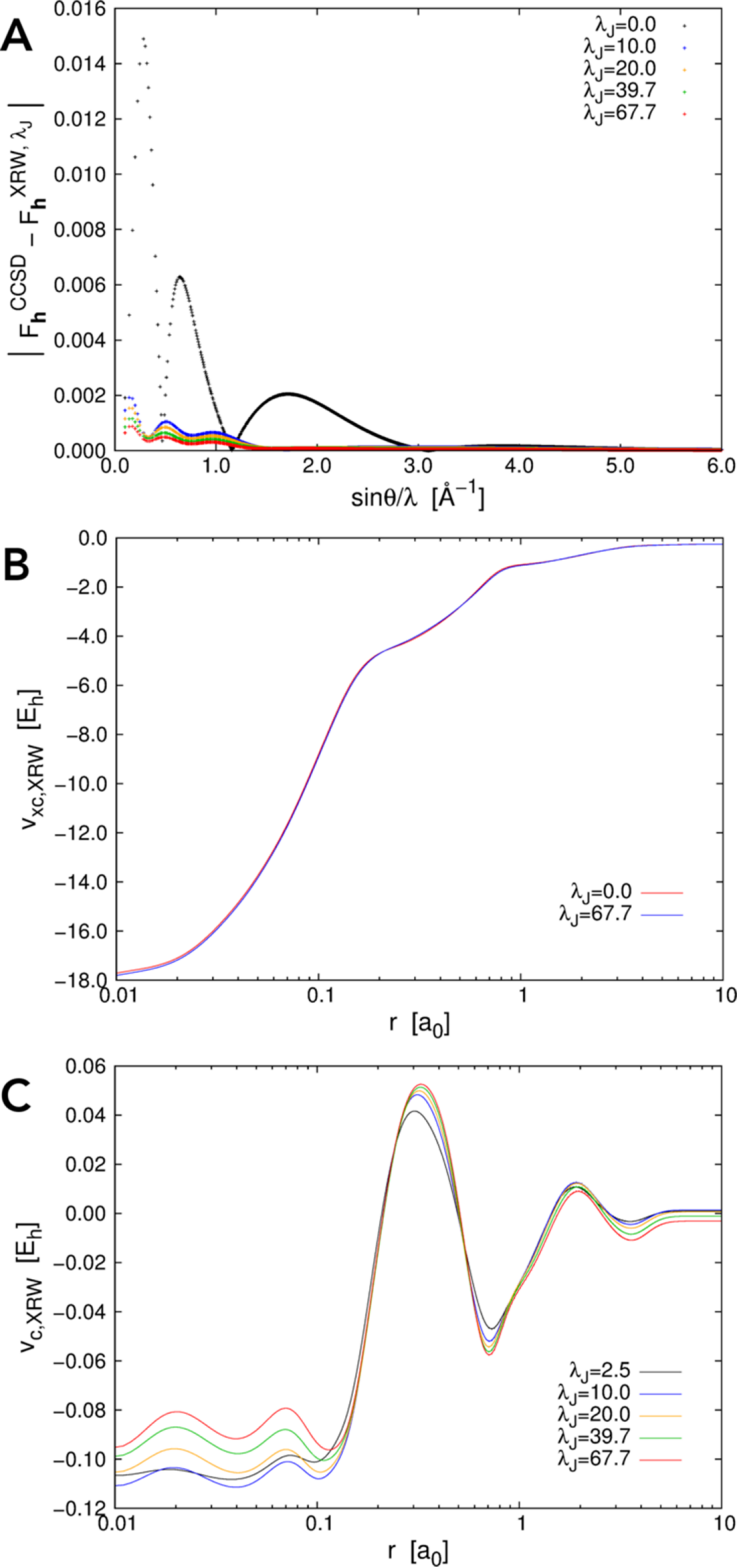
Argon: (*A*) absolute discrepancies between the XRW and CCSD/u6-311G* structure-factor amplitudes for different 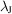 values, (*B*) orbital-averaged XRW xc potentials extracted for 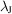 = 0.0 (Slater potential) and 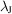
 = 67.7, and (*C*) orbital-averaged XRW perturbation potentials obtained at different 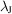 values.

**Figure 3:**
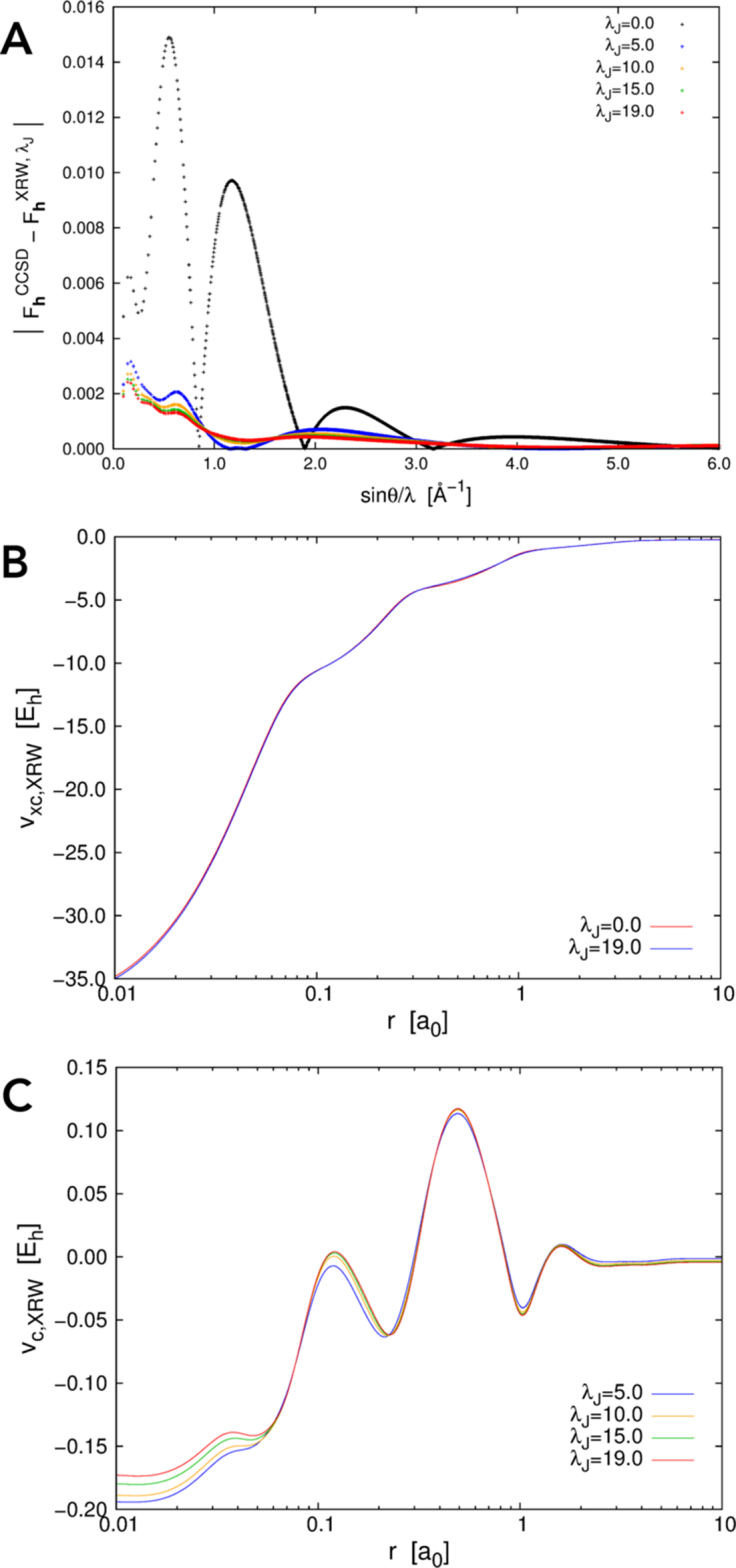
Krypton: (*A*) absolute discrepancies between the XRW and CCSD/u6-311G* structure-factor amplitudes for different 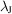 values, (*B*) orbital-averaged XRW xc potentials extracted for 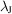 = 0.0 (Slater potential) and 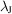
 = 19.0, and (*C*) orbital-averaged XRW perturbation potentials obtained at different 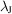 values.

Let us now consider the other two atomic systems, argon and krypton. Also in these cases, the discrepancies between the XRW and target CCSD structure-factor amplitudes (and consequently the discrepancy between the XRW and CCSD electron densities) overall decrease on increasing the value of the external parameter 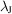. This emerges quite clearly from Figs. 2 ▸(*A*) and 3 ▸(*A*), from Table 2 ▸, and from Figs. S1(*B*) and S1(*C*). However, the goal of the XRW method is to improve the overall agreement between calculated and target X-ray data and not to improve the agreement for each single reflection. For this reason, for some structure-factor amplitudes, the discrepancy may increase when 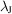 becomes larger [for example, see Fig. 3 ▸(*A*) concerning the krypton case].

The orbital-averaged XRW xc potentials for argon and krypton are reported in Figs. 2 ▸(*B*) and 3 ▸(*B*), respectively, where we depict the Slater potentials (namely, the orbital-averaged xc potentials extracted for 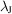 = 0.0, which practically correspond to potentials describing the pure exchange contributions in the Hartree–Fock regime) and the potentials corresponding to the maximum value of 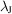 for which the XRW computations were performed. Like for neon, the xc potentials corresponding to the different 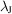 values are very similar [see also Figs. S2(*B*) and S2(*C*) for the graphs illustrating the differences between the potentials], further confirming the small size of the perturbation introduced in the calculations through the X-ray data. However, to better appreciate the differences, we can consider the orbital-averaged XRW perturbation potentials shown in Figs. 2 ▸(*C*) and 3 ▸(*C*), which, also in these cases, are very similar to the difference plots reported in Figs. S2(*B*) and S2(*C*) (always with the same caveat as pointed out for neon). Through the analyses of the perturbation potentials, we can observe that, for both argon and krypton, at small distances from the nucleus (until ∼0.08–0.1 Å) we have oscillations and/or a slower convergence to a well defined shape. This is again ascribable to the limited resolution of the X-ray diffraction data used in the XRW calculations. In these cases, the oscillations stop at a distance closer to the nucleus compared with what was observed for the orbital-averaged XRW perturbation potential of neon. This can be explained by the higher maximum resolution considered for argon and krypton. Finally, for distances from the nucleus larger than 0.08–0.1 Å, convergence is generally faster, although it decreases to a lower extent for very large *r* values due to the much lower number of reflections in the low-angle domain [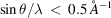; see the reduced number of points in this resolution range in Figs. 2 ▸(*A*) and 3 ▸(*A*) for argon and krypton, respectively].

Similar trends can also be observed for the Li_2_ system. First, we notice a progressive increase of the overall agreement between the XRW and target structure-factor amplitudes [see Figs. S1(*D*) and S3] and, consequently, also between the XRW and target electron distributions (see Table 3 ▸).

**Figure 4:**
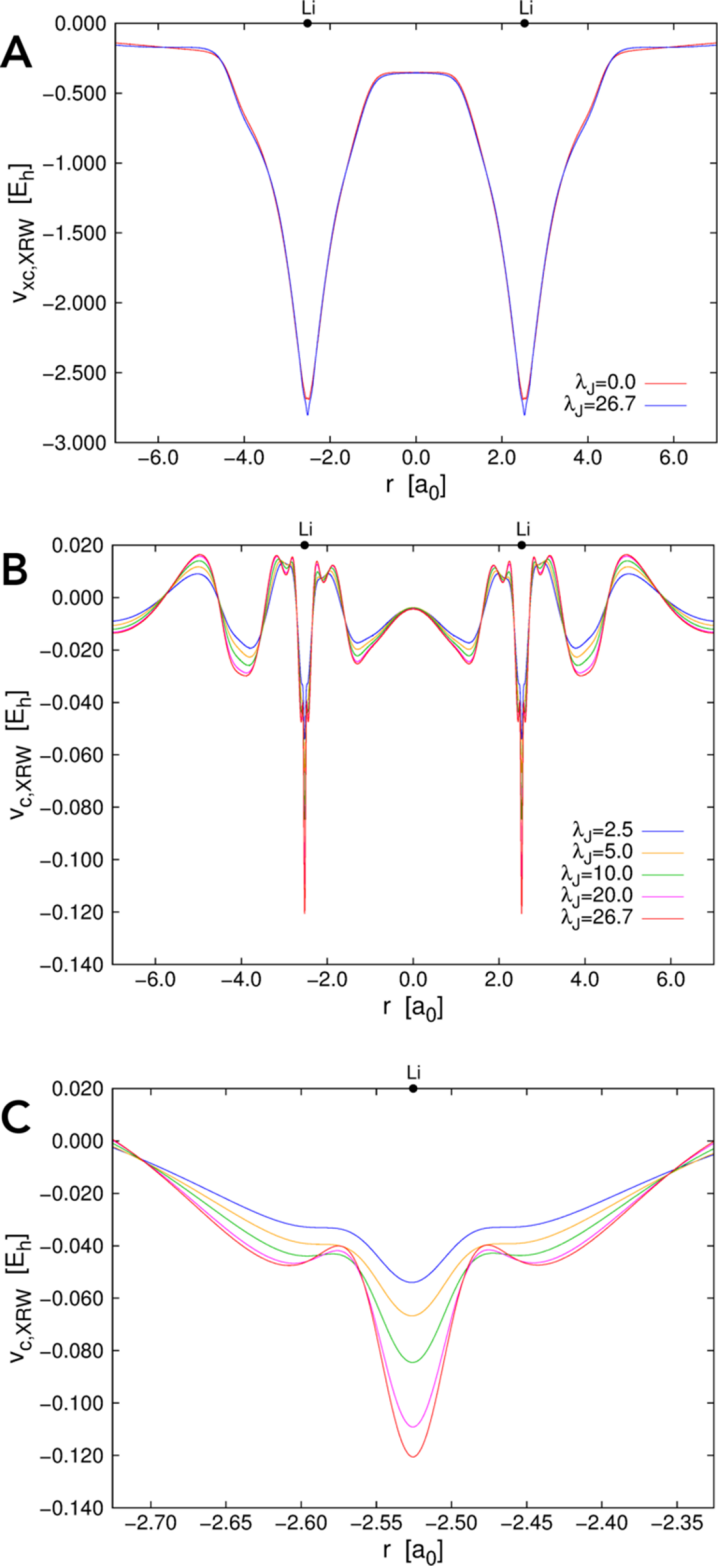
Dilithium: (*A*) orbital-averaged XRW xc potentials extracted for 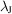 = 0.0 (Slater potential) and 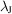 = 26.7, (*B*) orbital-averaged XRW perturbation potentials obtained at different 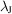 values, and (*C*) a zoomed-in view of the orbital-averaged XRW perturbation potentials obtained at different 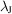 values around one of the two lithium nuclei.

Fig. 4 ▸(*A*) illustrates two orbital-averaged XRW xc potentials for Li_2_: one corresponding to 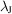
 = 0.0 (the Slater potential) and the other to 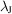
 = 26.7. Similar to the previously analysed atomic systems, the two potentials exhibit only minor differences. However, the Slater potential shows noticeable oscillations near the nuclei, which can be attributed to the partial failure of the smoothing procedure used for xc potentials derived from Gaussian basis sets (Gaiduk *et al.*, 2013 ▸; Kananenka *et al.*, 2013 ▸). In contrast, the procedure worked effectively for the potential at 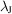 = 26.7, resulting in a smooth curve.

The subtle differences between the orbital-averaged XRW xc potentials for Li_2_ are more clearly discerned by examining the orbital-averaged XRW perturbation potentials, which are shown in Fig. 4 ▸(*B*). As with the atomic cases, oscillations are observed near the nuclear centres [see also the zoomed-in view around one of the nuclei in Fig. 4 ▸(*C*)], which can be attributed to the finite resolution of the X-ray diffraction data employed in the XRW calculations. In particular, in agreement with the maximum resolution considered in this case (*i.e.* 4.0 Å^−1^) and with Bragg’s law, the oscillations occur until about 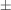
0.125 Å from the nuclei [see again Fig. 4 ▸(*C*)]. The situation improves at greater distances from the nuclei, particularly in the bonding region, where the perturbation potentials gradually converge to a well defined shape as the external parameter 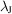 increases. This is the result of introducing the perturbation of the X-ray structure-factor amplitudes through the XRW calculations.

**Figure 5:**
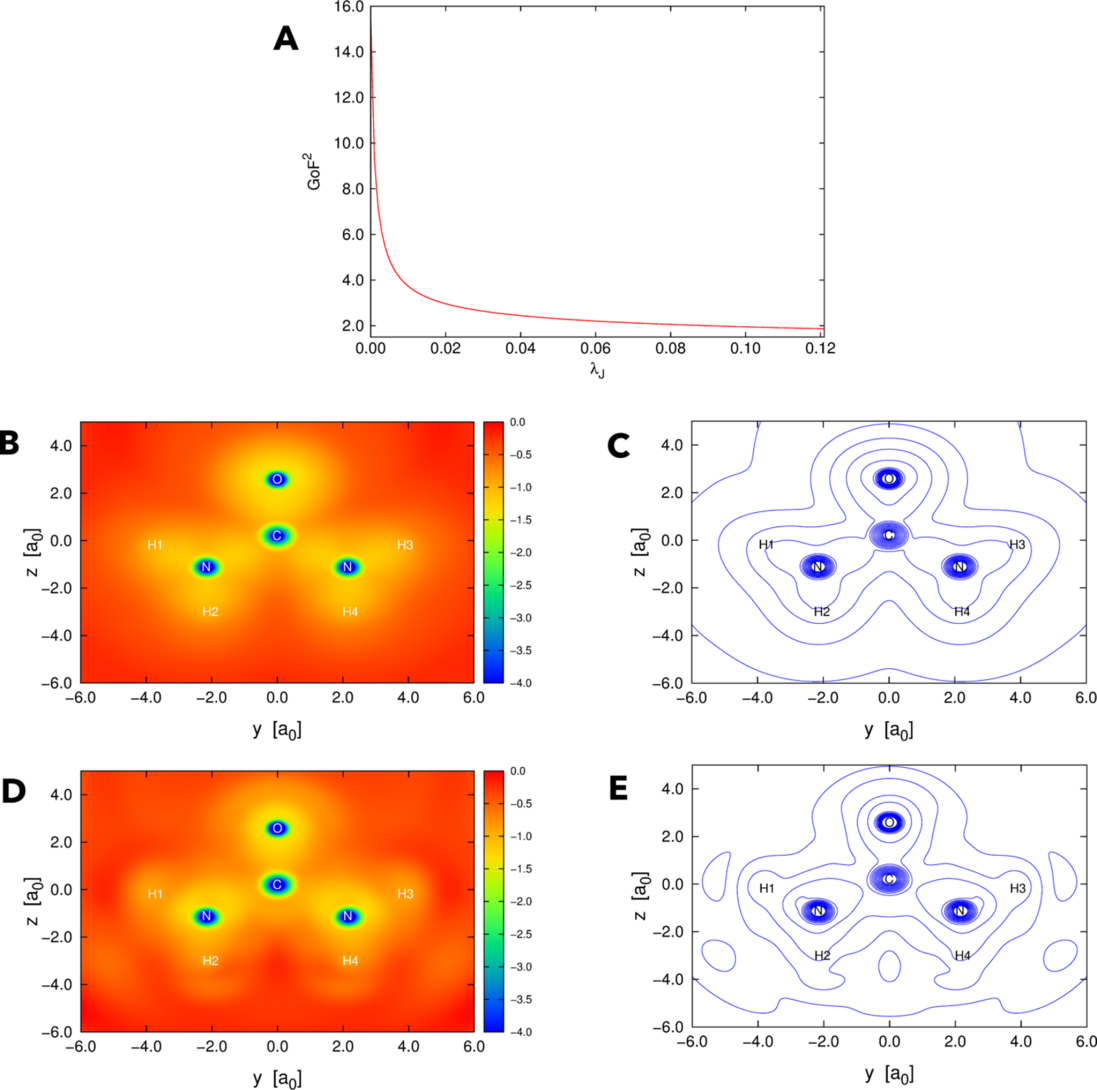
Urea with experimental X-ray data: (*A*) GoF^2^ as a function of 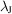, (*B*) orbital-averaged XRW xc potentials extracted for 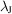 = 0.0 (Slater potential) represented as a heat map, (*C*) orbital-averaged XRW xc potentials extracted for 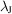 = 0.0 (Slater potential) represented with isolines, (*D*) orbital-averaged XRW xc potentials extracted for 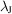 = 0.121 represented as a heat map, and (*E*) orbital-averaged XRW xc potentials extracted for 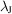 = 0.121 represented with isolines. Both in the heat maps and in the graphs with isolines, the xc potentials are represented in the plane of the urea molecule in its in-crystal geometry. In the heat maps, the values of the potentials are in Hartree (E_h_), and the values lower than or equal to −4.0 E_h_ are depicted in dark blue. In the graphs with isolines, the isocontours range from −4.0 E_h_ to −0.25 E_h_ with steps of 0.25 E_h_.

So far, we have considered only orbital-averaged XRW potentials corresponding to theoretically generated X-ray diffraction data. We will now analyse the results obtained with the high-resolution and high-quality X-ray data collected for the urea crystal. First of all, as for the XRW computations with theoretical structure-factor amplitudes, the agreement between theoretical and target data gradually increases with 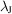. This is clearly observable in Fig. 5 ▸(*A*) where GoF^2^ [see equation (3)] monotonically decreases as a function of 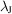.

The orbital-averaged XRW xc potentials for 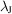 = 0.0 (Slater potential) and 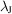 = 0.121 (the maximum value of the external parameter) are shown in Figs. 5 ▸(*B*), 5 ▸(*C*), 5 ▸(*D*) and 5 ▸(*E*) as heat maps or graphs with isolines in the plane of the urea molecule in its planar experimental in-crystal geometry. Even with experimental data as restraints, the orbital-averaged xc potentials remain very similar. Only slight differences can be observed: when the X-ray structure factors are considered, the xc potential becomes less negative around the carbon and oxygen nuclei and nearly vanishes in the outer regions. In the xc potential for 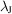
 = 0.121, there is a quasi-separation into atomic domains.

**Figure 6:**
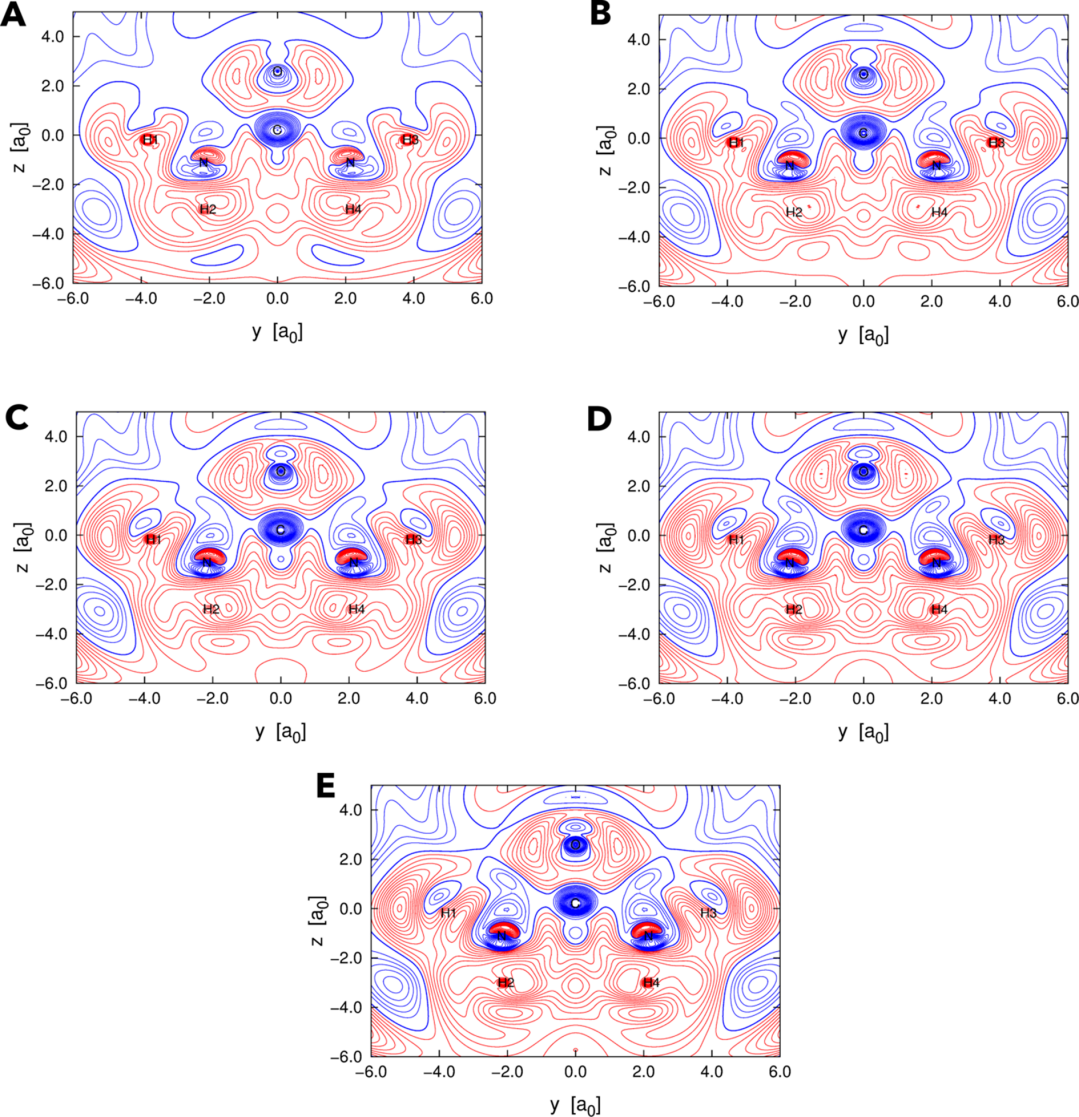
Orbital-averaged XRW perturbation potentials resulting from XRW calculations on urea with experimental X-ray diffraction data. The potentials are represented as graphs with isolines in the plane of the urea molecule in its planar in-crystal geometry for different values of 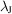: (*A*) 0.025, (*B*) 0.050, (*C*) 0.075, (*D*) 0.100 and (*E*) 0.121. The isocontours extend from −0.5 E_h_ to 0.3 E_h_, with intervals of 0.025 E_h_. Negative and positive isocontours are represented in blue and red, respectively, while the isocontour corresponding to 0.0 E_h_ is distinguished by a thicker blue line.

Let us now examine the extracted orbital-averaged XRW perturbation potentials, shown in Figs. 6 ▸ and S4 for various values of 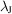 (0.025, 0.050, 0.075, 0.100 and 0.121). As the external parameter increases, the potentials gradually converge to a well defined structure. Specifically, the perturbation potential in the vicinity of the carbon and oxygen nuclei becomes progressively more negative, whereas the nitro­gen site is encompassed by both negative and positive regions. The behaviour in the vicinity of the hydrogen atoms differs: for hydrogen atoms H1 and H3, the potential gradually diminishes, whereas for H2 and H4, convergence is slightly mis-ordered with respect to 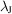, although the potential ultimately becomes more positive. In outer domains, such as in the region of the oxygen lone pairs or at greater distances from the hydrogen nuclei, the potential becomes progressively more positive.

**Figure 7:**
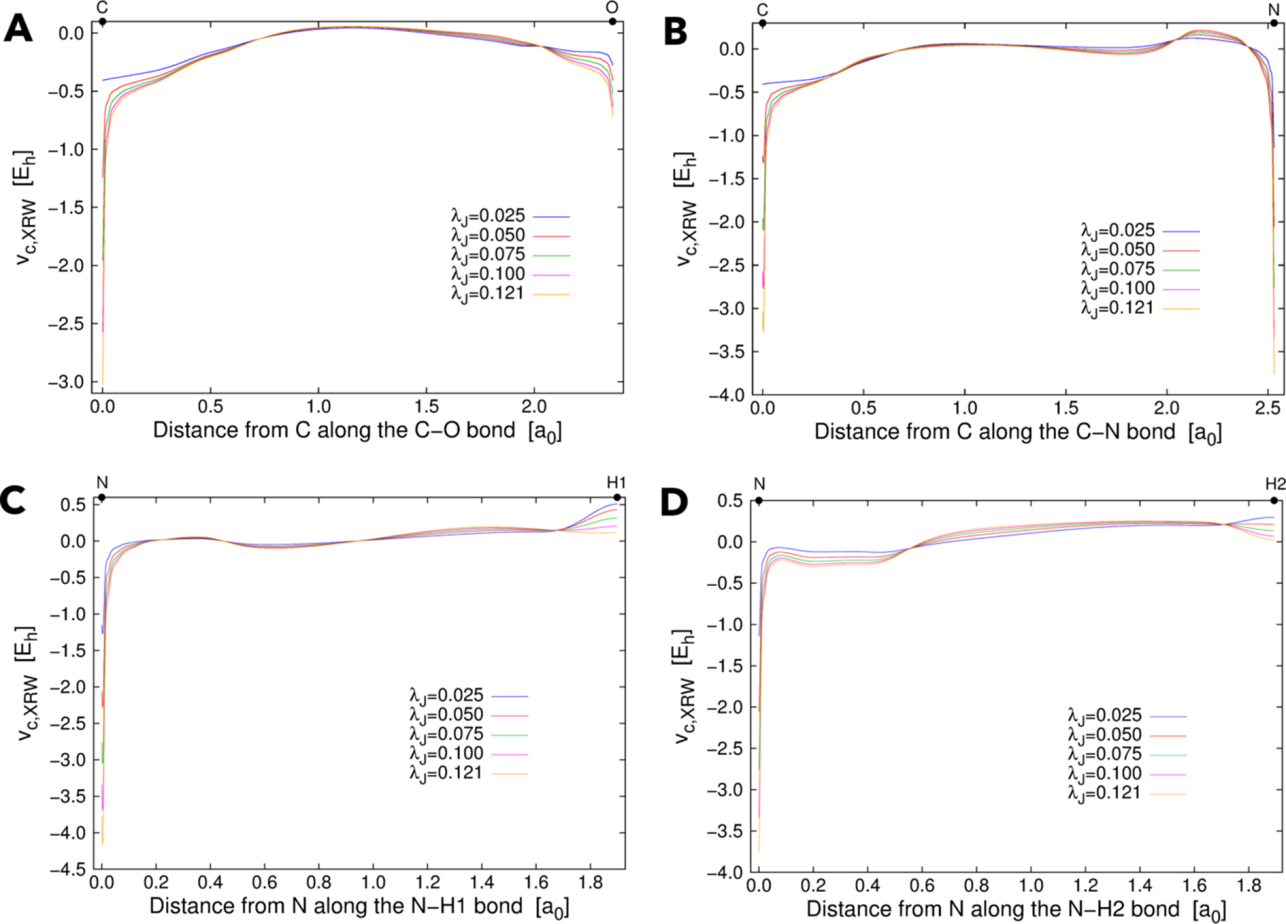
Orbital-averaged XRW perturbation potentials resulting from XRW calculations on urea with experimental X-ray diffraction data and with different 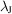 values. The potentials are represented along the (*A*) C–O, (*B*) C–N, (*C*) N–H1 and (*D*) N–H2 bonds of urea.

For completeness, Fig. 7 ▸ also shows the orbital-averaged XRW perturbation potentials obtained along several well defined bonds in urea. Along the C–O bond [see Fig. 7 ▸(*A*)], the extracted potentials are lowest at the positions of the carbon and oxygen nuclei and reach a relative maximum near the midpoint of the bond distance. The perturbation potentials are lowest at the atomic nuclei also along the C–N bond [see Fig. 7 ▸(*B*)]; however, in this case, two maxima are observed: one near the carbon atom and the other near the nitro­gen atom. The behaviour is different for the N–H1 and N–H2 bonds [see Figs. 7 ▸(*C*) and 7 ▸(*D*), respectively], with potentials that are lowest at the nitro­gen nucleus and that progressively increase with oscillations along the N–H bonds. Also in Fig. 7 ▸ we can observe a sort of convergence as the value of the external parameter 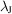 increases.

However, as noted in the discussion of the theoretical test cases, also in the case of urea the maximum resolution actually limits the accurate modelling of the potentials close to the atomic nuclei. For the X-ray diffraction data collected on the urea crystal, the maximum resolution is 1.44 Å^−1^, much lower than in the theoretical cases discussed earlier. Therefore, by again using Bragg’s law, this means that the available structure factors cannot be used to accurately model exchange–correlation or perturbation potentials at distances lower than 0.347 Å from the nuclei.

## 4. Conclusions

In this paper, prompted by the inherent ability of the Jayatilaka approach in capturing electron correlation and polarization effects on electron distributions (Genoni, Dos Santos *et al.*, 2017 ▸; Ernst *et al.*, 2020 ▸; Hupf *et al.*, 2023 ▸), and in providing consistent electron densities (Genoni & Martín Pendás, 2024 ▸), we have shown and discussed for the first time the perturbation potentials that are involved in XRW calculations. This was achieved by extending to the XRW method an inversion procedure previously applied in DFT to determine orbital-averaged exchange–correlation and correlation potentials (Kananenka *et al.*, 2013 ▸). The technique is applicable with both theoretically generated X-ray structure-factor amplitudes and high-quality experimental X-ray diffraction data.

Although the potentials extracted from XRW calculations are currently limited to orbital-averaged potentials, we have already identified distinct features that may be representative of real potentials. These features underscore both the potential benefits and limitations of using X-ray data, and particularly the XRW approach, for developing new xc functionals. Notably, we consistently observed that the Slater potentials and orbital-averaged potentials from XRW calculations are quite similar, suggesting that the perturbation introduced by the X-ray data is minimal. Additionally, the orbital-averaged perturbation potentials display noticeable oscillations near the nuclei, extending up to a distance determined by the maximum resolution of the X-ray data according to Bragg’s law. This implies that the resolution actually limits the degree to which X-ray structure factors can be applied to model exchange–correlation and correlation potentials: while X-ray data can effectively improve potentials (and thus functionals) in the valence regions, they are currently inadequate for refining potentials in the atomic core regions. In this context, it is also important to consider the significantly lower resolutions achievable through X-ray diffraction experiments (even in accurate charge-density studies like those on the urea crystal considered in this work) compared with theoretical cases. Finally, we also observed that a lower number of low-angle reflections generally leads to slower convergence toward the final structure of the potentials in the outermost regions of space. This is another point to consider when using X-ray diffraction data to develop or refine xc functionals.

The research work presented in this paper is only the first preliminary step towards the possible exploitation of the XRW strategy in the development of new xc functionals. Bearing in mind the above-mentioned limitations, some follow-up studies can be already envisaged. For example, it could be interesting to apply the XRW inversion procedure introduced in this paper to also analyse how and to what extent xc potentials corresponding to already existing xc functionals are perturbed when X-ray restrained DFT calculations are performed. Additionally, as discussed in Section 2.2, it will be essential to overcome the intrinsic limitations of the XRW inversion method, which currently yields only orbital-averaged potentials. This could be achieved by integrating the Jayatilaka strategy with the Ryabinkin–Kohout–Staroverov technique (Ryabinkin *et al.*, 2015 ▸; Cuevas-Saavedra *et al.*, 2015 ▸) or its variant (Ospadov *et al.*, 2017 ▸), enabling the extraction of more accurate XRW exchange–correlation and perturbation potentials that will be free from the shortcomings typically associated with density-to-potential mapping approaches. Reliable XRW xc potentials obtained in this way could then be possibly used as ingredients or models to propose new functionals, even exploiting machine-learning strategies. Finally, one could also imagine exploiting the XRW method in its DFT version to refit already existing xc functionals in order to finally obtain functionals fully compatible with accurate experimental or high-level theoretical electron densities.

In conclusion, we hope that the investigation presented in this paper could serve as a foundation for future efforts to apply the Jayatilaka method in developing new xc functionals. Despite current limitations imposed by the resolution of X-ray data used in the calculations, we believe that the inherent features and strengths of the XRW technique make it a promising tool for proposing functionals that align with the recommendations recently emphasized by Medvedev *et al.* (2017 ▸), staying close to the original principles and spirit of DFT.

## Supplementary Material

Supporting information. DOI: 10.1107/S1600576725004765/pen5008sup1.pdf

## Figures and Tables

**Table 1:** Details about the computation of theoretical X-ray structure-factor amplitudes

System	Level of theory and basis set	Cubic unit-cell edge (Å)	Max. resolution (Å^−1^)	Total No. of reflections
Ne	CCSD/UGBS	10	2.0	133880
Ar	CCSD/u6-311G*	5	6.0	452044
Kr	CCSD/u6-311G*	5	6.0	452044
Li_2_	CCSD/u6-311G*	7.5	4.0	452044

**Table 2:** Global absolute errors in the density (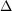) at different 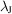 values for the XRW calculations on neon with CCSD/UGBS structure-factor amplitudes, on argon with CCSD/u6-311G* structure-factor amplitudes and on krypton with CCSD/u6-311G* structure-factor amplitudes

Neon–CCSD/UGBS		Argon–CCSD/u6-311G*		Krypton–CCSD/u6-311G*	
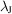	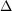 (e)	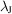	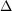 (e)	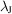	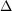 (e)
0.0	0.1194	0.0	0.0678	0.0	0.0916
10.0	0.0044	10.0	0.0070	5.0	0.0185
40.0	0.0015	20.0	0.0054	10.0	0.0080
80.0	0.0010	40.0	0.0039	15.0	0.0066
120.0	0.0008	67.7	0.0030	19.0	0.0060

**Table 3:** Global absolute errors in the density 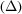 at different 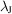 values for the XRW calculations on Li_2_ with CCSD/u6-311G* structure-factor amplitudes

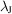	0.0	2.5	5.0	10.0	20.0	26.7
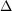 (e)	0.0700	0.0145	0.0126	0.0116	0.0111	0.0110

## Data Availability

Data supporting the results are available upon reasonable request to the corresponding author of this paper.

## Footnotes

1 The notation  has been adopted to distinguish the typical external parameter of the XRW approach from the radiation wavelength, which will appear in other sections of the text and will be simply indicated with .

2 We used  instead of , in agreement with the terminology proposed by Davidson *et al.* (2022*a* ▸,*b* ▸).
