# Supplementary material for: Inversion of the X-ray restrained wavefunction equations: a first step towards the development of exchange–correlation functionals based on X-ray data
Source: J Appl Crystallogr. 2025 Jul 25;58(Pt 4):1106–21. doi: 10.1107/S1600576725004765 (PMC12321031; doi:10.1107/S1600576725004765)
Supplement: Supplementary file 1 [file j-58-01106-sup1.pdf]

JOURNAL OF APPLIED CRYSTALLOGRAPHY

Volume 58 (2025)

Supporting information for article:

Inversion of the X-ray restrained wavefunction equations: a first step towards the development of exchange-correlation functionals based on X-ray data

Alessandro Genoni and Maurizio Sironi

S1. Weighting scheme for XRW calculations with theoretically generated data

When using theoretically generated X-ray diffraction data corresponding to a single molecule or atom in a large cubic unit-cell, we observe the following: (i) reflections are unevenly distributed across the resolution shells, with high-angle data being much more abundant than low- and medium-angle data; (ii) the discrepancies in structure factor amplitudes calculated at the unrestrained level (namely, at Hartree-Fock level) compared to reference values (namely, structure factor amplitudes derived from gas-phase correlated electron densities) are quite pronounced at low- and medium-angles but generally minimal at high-angles. Based on these observations, and considering the expression for the Goodness-of-Fit (GoF^2) in the functional to be minimized, as shown in Eqs. (2) and (3) of the main text, the predominance of high-angle data leads to a substantial down-weighting of the contributions from low- and medium-angle reflections when the high-angle data are fully considered.

Therefore, to try to make importance of all the structure factor amplitudes more uniform across the different resolution domains, the expression of the square of the Goodness-of-Fit (GoF^2) is modified as follows:

$$\mathrm{GoF}^2[\Psi_0] = \frac{1}{N_r - N_p} \sum_{\boldsymbol{h}} w_{\boldsymbol{h}} \frac{\left(\eta\, F_{\boldsymbol{h}}^{calc}[\Psi_0] - F_{\boldsymbol{h}}^{obs}\right)^2}{\sigma_{\boldsymbol{h}}^2} \qquad \text{(S1)}.$$

$w_{\boldsymbol{h}}$ is determined by taking into account the distribution of the reflections in the reciprocal space. In particular, for a reflection characterised by the Miller indices $\boldsymbol{h} = (h, k, l)$ and of resolution $\sin\theta_{\boldsymbol{h}}/\lambda$, its weight $w_{\boldsymbol{h}}$ is defined as the inverse of the "local density" ($\rho_{\boldsymbol{h}}$) of X-ray diffraction data at that resolution:

$$w_{\boldsymbol{h}} = \frac{1}{\rho_{\boldsymbol{h}}} \qquad \text{(S2)},$$

where $\rho_{\boldsymbol{h}}$ is

$$\rho_{\boldsymbol{h}} = \frac{n_{\boldsymbol{h}}}{N_r} \qquad \text{(S3)}.$$

In equation (S3), N_r is the total number of X-ray structure factor amplitudes employed in the XRW calculation and $n_{\boldsymbol{h}}$ the number of reflections in the interval $\left[\sin\theta_{\boldsymbol{h}}/\lambda - \frac{\Delta}{2},\ \sin\theta_{\boldsymbol{h}}/\lambda + \frac{\Delta}{2}\right]$. Δ is an empirical parameter that provides the width of the above-mentioned interval and whose value is chosen by the user before starting the computations (in this work, Δ always was always set equal to 0.010 Å^{-1} for the XRW calculations with theoretical structure factor amplitudes).

S2. Dilithium and urea geometries

Table S1 Li_2 geometry used in the calculations (coordinates in Å)

	X	Y	Z
Li	0.000000	0.000000	-1.336437
Li	0.000000	0.000000	1.336437

Table S2 Experimental in-crystal geometry of urea used in the calculations (coordinates in Å)

	X	Y	Z
C	0.00000000	2.78900000	1.53799206
O	0.00000000	2.78900000	2.79444924
N	0.80702504	3.59602504	0.83884086
H	1.42629460	4.21529460	1.33129260
H	0.79821180	3.58721180	-0.16307280
N	-0.80702504	1.98197496	0.83884086
H	-1.42629460	1.36270540	1.33129260
H	-0.79821180	1.99078820	-0.16307280

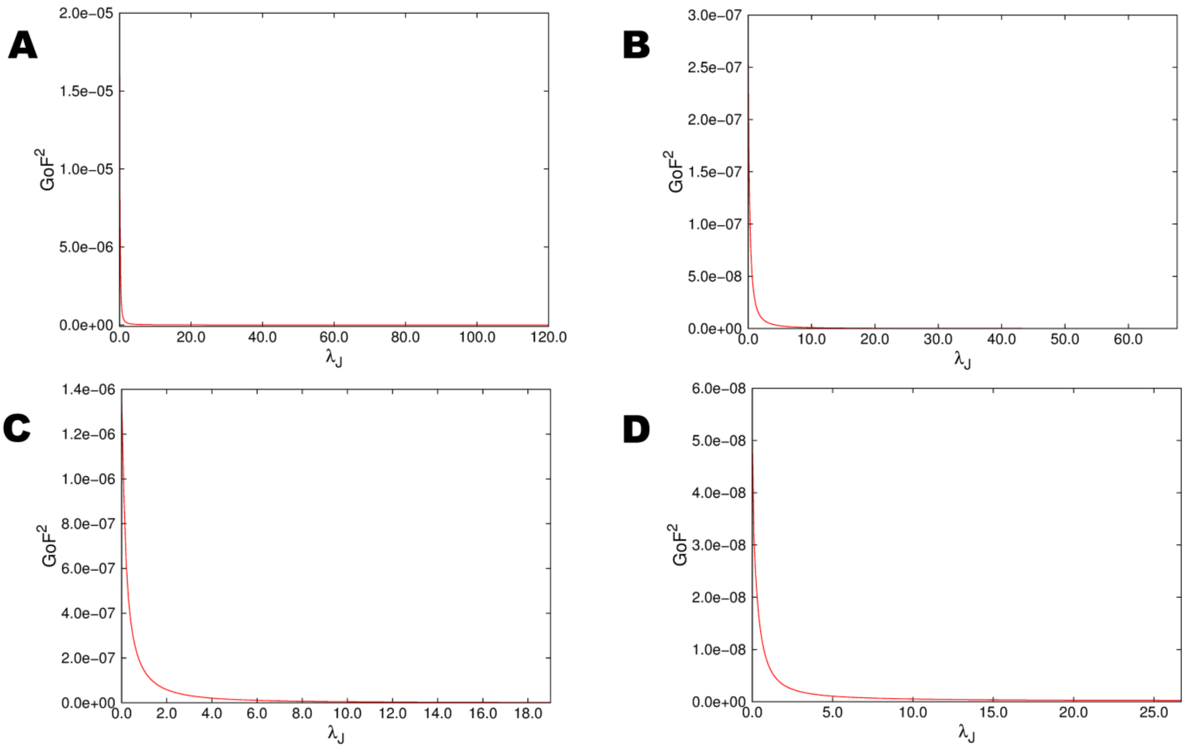

Figure S1 Square of the Goodness-of-Fit GoF^2 as a function of λ_J for the XRW calculations on: (A) neon with X-ray diffraction data theoretically generated at CCSD/UGBS level; (B) argon with X-ray diffraction data theoretically generated at CCSD/u6-311G* level; (C) krypton with X-ray diffraction data theoretically generated at CCSD/u6-311G* level; (D) Li_2 with X-ray diffraction data theoretically generated at CCSD/u6-311G* level.

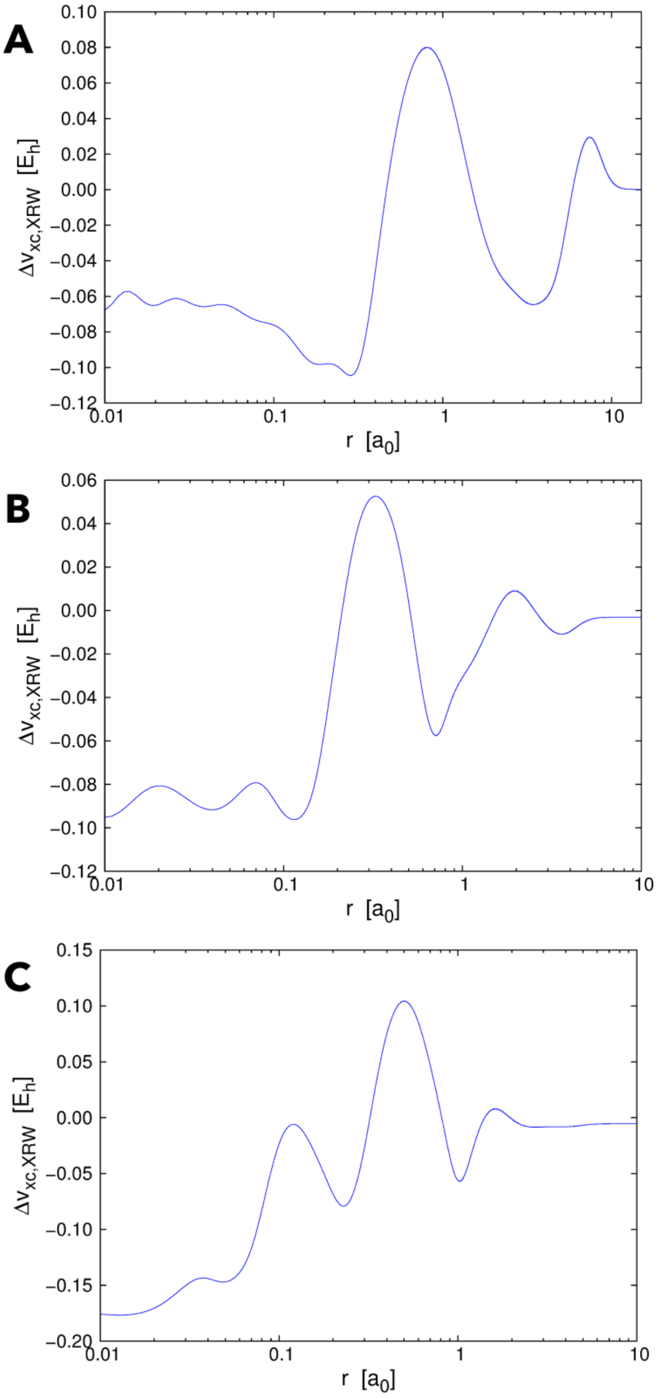

Figure S2 (A) Neon: difference between the orbital-averaged XRW xc-potentials extracted for λ_J = 120.0 and λ_J = 0 (Slater potential); (B) argon: difference between the orbital-averaged XRW xc-potentials extracted for λ_J = 67.7 and λ_J = 0 (Slater potential); (C) krypton: difference between the orbital-averaged XRW xc-potentials extracted for λ_J = 19.0 and λ_J = 0 (Slater potential).

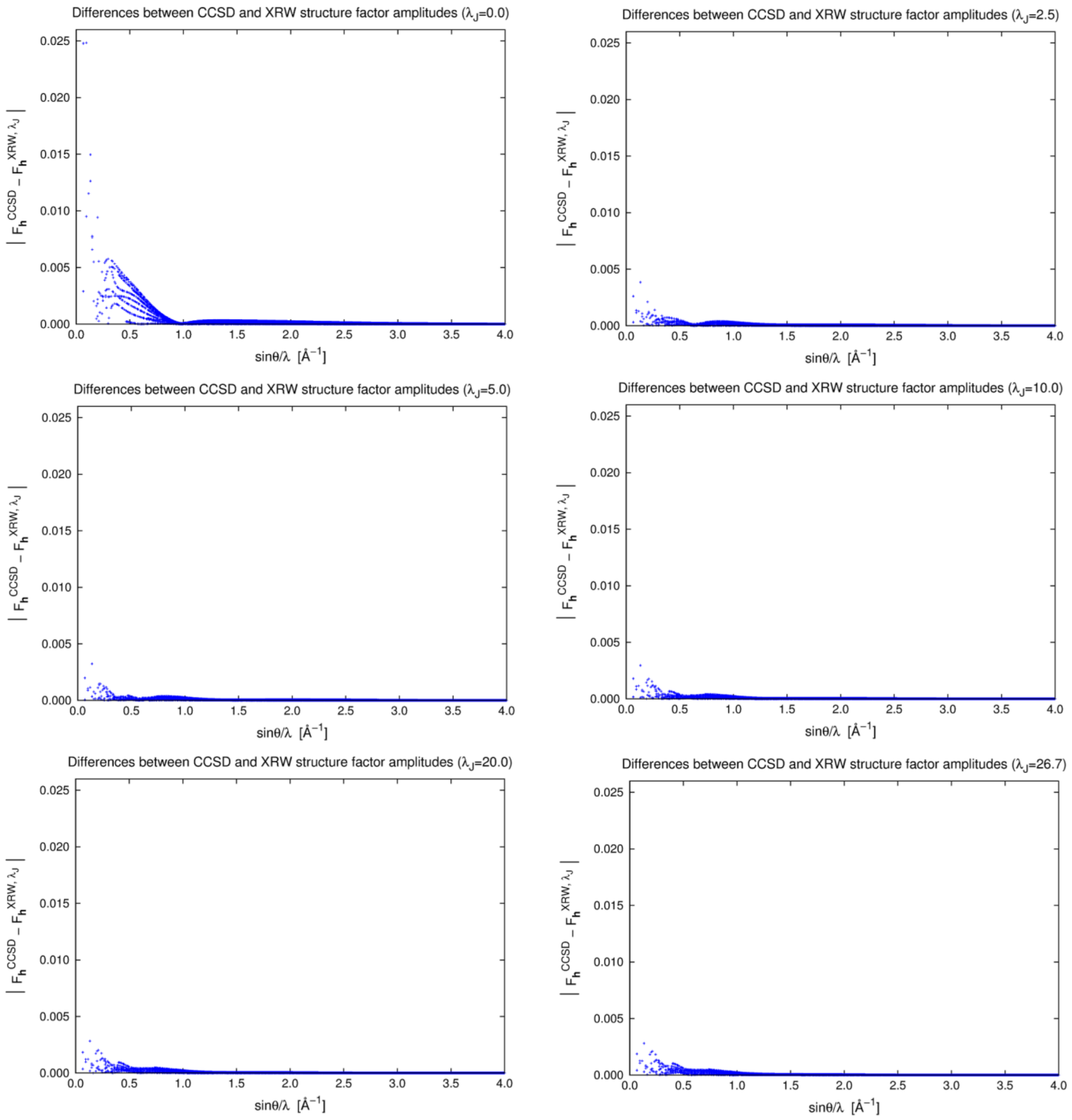

Figure S3 XRW calculations on Li_2: absolute discrepancies between the XRW and CCSD/u6-311G* structure factor amplitudes for different λ_J values.

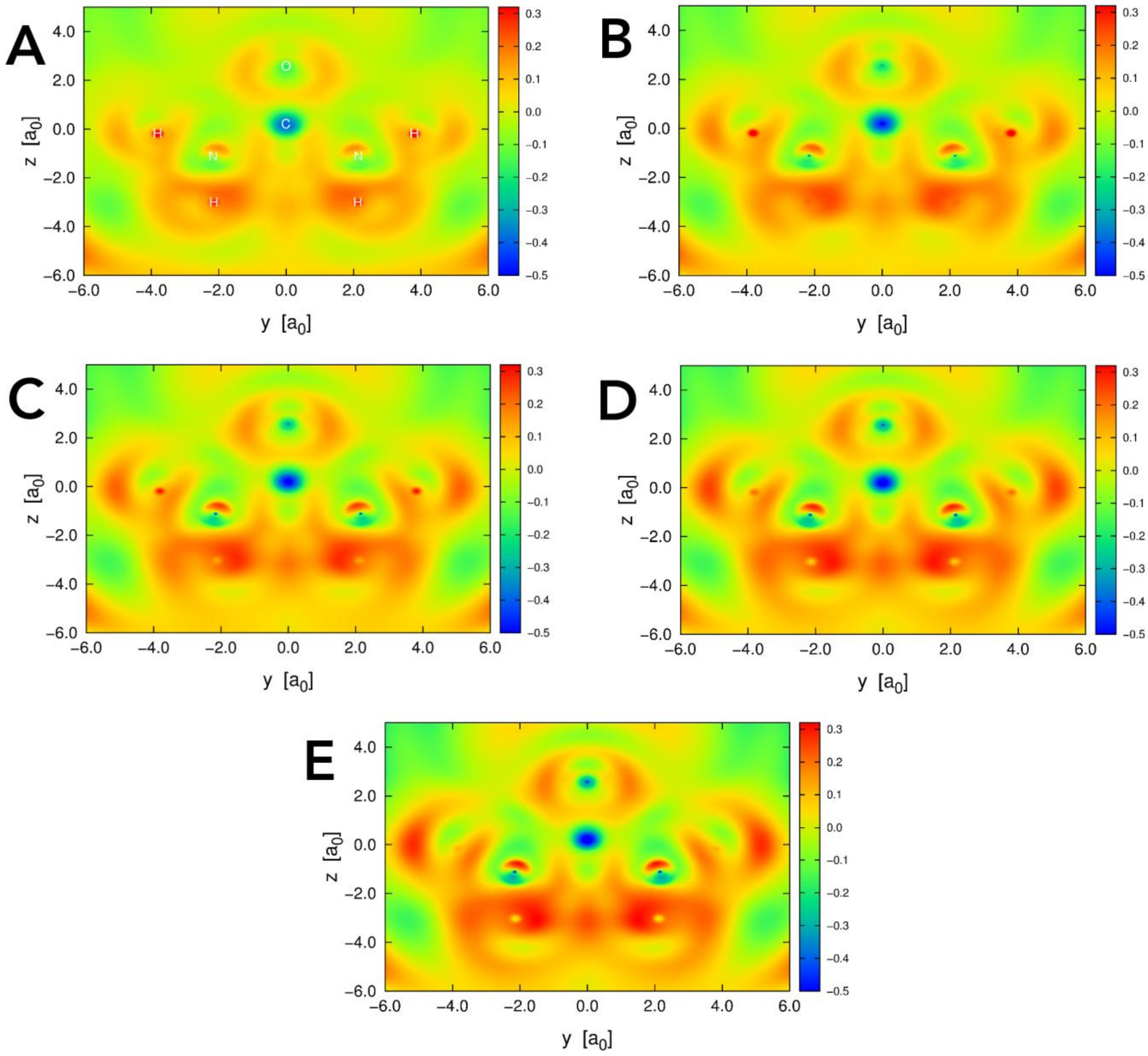

Figure S4 Orbital-averaged XRW perturbation potentials resulting from XRW calculations on urea with experimental X-ray diffraction data. The potentials are represented as heat-maps on the plane of the urea molecule in its planar in-crystal geometry for different values of λ_J: (A) 0.25, (B), 0.50, (C) 0.75, (D) 0.100, and (E) 0.121. The values of the potentials are in Hartree (E_h).
